# Integrated metabolomic and transcriptomic analyses identify critical genes in eicosapentaenoic acid biosynthesis and metabolism in the sea urchin *Strongylocentrotus intermedius*

**DOI:** 10.1038/s41598-020-58643-x

**Published:** 2020-02-03

**Authors:** Heng Wang, Jun Ding, Siyu Ding, Yaqing Chang

**Affiliations:** Key Laboratory of Mariculture & Stock Enhancement in North China’s Sea, Ministry of Agriculture and Rural Affairs, Dalian Ocean University, Dalian, 116023 China

**Keywords:** Gene expression, Transcriptomics

## Abstract

Gonads are the only edible part of the sea urchin and have great potential as a health-promoting food for human consumption. Polyunsaturated fatty acids (PUFAs) are important necessary nutrients that determine not only the nutritional value of sea urchins but guarantee their normal growth and reproduction. However, the information on the molecular mechanisms of PUFA biosynthesis and metabolism in this species remains elusive. In this study, we used *Strongylocentrotus intermedius* as our model species and conducted integrated metabolomic and transcriptomic analyses of potentially critical genes involved in PUFA biosynthesis and metabolism during gonad growth and development, mainly focusing on eicosapentaenoic acid (EPA). We found six differentially accumulated metabolites associated with PUFA in the metabolomic analysis. More differentially expressed genes (DEGs) were related to PUFA in testis than ovary (1823 DEGs in testis and 1499 DEGs in ovary). We verified 12 DEGs by RNA-Seq results and found that Aldh7a1, Ecm3, Fads2, and Hsd17b12 genes had similar expression patterns in EPA concentration during gonad growth and development. In contrast, the other DEGs were downregulated and we inferred that EPA or PUFA may be metabolized as energy during certain periods. Our metabolic and genetic data will facilitate a better understanding of PUFA regulation networks during gonad growth and development in *S. intermedius*.

## Introduction

The gonads which produce roe are the only edible part of the sea urchins and have great potential to promote benfites to human health^1,2^. The sea urchin gonads have high nutritional value and possess several essential nutrients like protein, lipids, polysaccharides, fatty acids, minierlas and vitamins. Polyunsaturated fatty acids (PUFAs) are one of‘ the most important and necessary nutrients, with eicosapentaenoic acid (EPA) as the main component among the PUFAs. Many biophysical studies have revealed that PUFAs significantly alter the basic properties of membranes such as acyl chain order, fluidity, elastic compressibility, phase behavior and permeability^3,4^, and play a beneficial role in stabilizing dynamic membrane^5^, membrane organization and cell division^6^. In addition, some PUFAs have significant influence on the production of eicosanoids, which involved in the body’s inflammatory response and homeostatic processes, neurological disorders, and cardiovascular diseases^7,8^. The potential of PUFA in stimulating bone, brain and immune cell development at embryonic through to early phases of the animal’s life could significantly improve productivity and welfare^9^. In marine invertebrates, PUFAs play a key role in metabolic health and influence various cellular processes, such as serving as a repository for energy reserves or regulating gene expression of metabolic disorders^10^.

It is believed that PUFAs could be synthesized in marine invertebrates^11^, such as mollusks, in which the biosynthesis of PUFA has been most extensively investigated^12^. However, unlike vertebrates, information on biosynthetic pathways and the molecular mechanism of PUFA biosynthesis and metabolism in marine invertebrates are still limited. Therefore, studying PUFA biosynthetic pathways and the molecular mechanism of PUFA biosynthesis may provide novel insights into fatty acid biosynthesis and metabolism in invertebrates, and may alter or enhance more efficient PUFA production for human nutrition.

The application of modern molecular genetic techniques and omics research (i.e., genomics, proteomics, transcriptomics, metabolomics, epigenomics, etc.) are further increasing the understanding of the cascades from genes to phenotypes^13^. However, a single omics research provides limited insight into the complex molecular pathways^14^. Integrated omics approaches are introduced to provide a comprehensive understanding the complexity of biological systems via interpretations using bioinformatics analyses^15,16^. For instance, the transcriptomes throughout embryonic development of *Strongylocentrotus purpuratus* using RNA-seq were analyzed to understand the global gene regulatory networks during the embryonic development of sea urchins^17^; a comparative transcriptome analysis of different colored tube feet in *Strongylocentrotus intermedius* was conducted to understand the differences at the molecular level and provide valuable information for marker-assisted breeding in this species^18^; nuclear magnetic resonance (NMR)-based metabolomics was applied to embryos of the sea urchin *Arbacia lixula* to reveal the effects of decreased levels of choline and N-acetyl serotonin on the cholinergic and serotoninergic system^19^; the metabolomic and de novo transcriptomic methods were used to investigate the reproductive success of the sea urchin *Paracentrotus lividus* which fed on two abundantly occurring benthic diatoms^20^. The integration of both metabolomics and transcriptomics is a powerful tool for revealing the biosynthetic mechanisms of key metabolic pathways^21^. Therefore, such studies can be used as an initial step to investigate the critical genes and the process of PUFA biosynthesis and metabolism in the sea urchin.

*Strongylocentrotus intermedius*, a cold-water species, is a commercially important sea urchin that is distributed along the Pacific coastal waters of Japan, Korean, and northeastern China^22^. *S. intermedius* originated on the coast of Hokkaido, Japan, and it was introduced to China in 1989. And now this species has become one of the most harvested sea urchins in China^23,24^. However, it is difficult to fully understand the mechanism of biosynthesis and metabolism of PUFAs due to the lack of genomic information and comprehensive analysis for this species. Moreover, which are the differentially expressed genes (DEGs), how to regulate metabolic pathways of PUFA in sea urchin, and how to modulate various metabolites of PUFA are still largely unknown.

In this study, we used an untargeted metabolomics and transcriptomics approach and an integrated analysis was performed. Based on the metabolomic and transcriptomic information of both sexes, the metabolic pathway related to PUFA biosynthesis and metabolism in *S. intermedius* were revealed in detail by the corresponding genes and intermediary metabolites, in particular, EPA related genes expression was analyzed in this study. The important metabolic and genetic information will conduce to better understand the characteristics and the regulatory networks of PUFAs during gonad growth and development in *S. intermedius* and could form a basis for further study to regulate growth traits in this species.

## Results

### Sea urchin body size and gonad development

**Figure 1:**
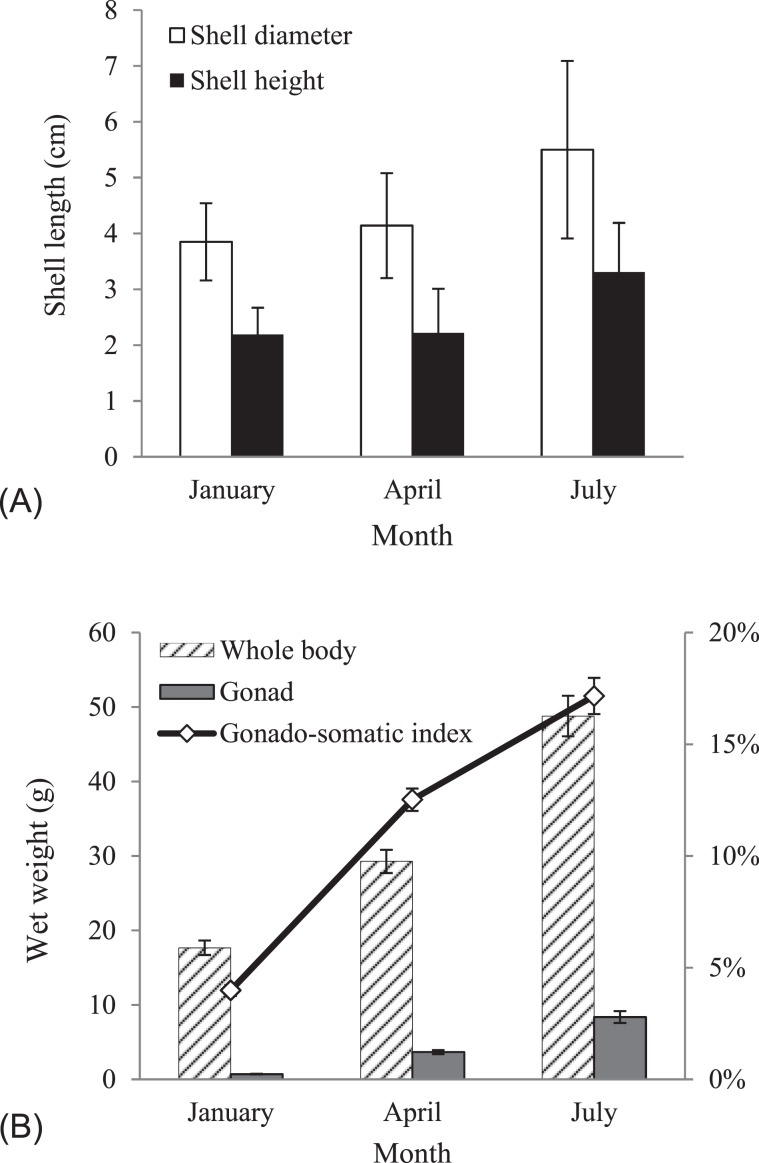
Changes of body size and weight in the sea urchin *Strongylocentrotus intermedius*, which was sampled for RNA and metabolite extraction. (**A**) Shell size; (**B**) wet weight and gonad somatic index (GSI) of sea urchin changes during gonad development stages. The results are expressed as mean ± SEM (n = 6).

The experiment lasted for 7 months from January 2018 to July 2018. A significant increase in shell diameters, shell height, wet body weight and gonad weight of *S. intermedius* was observed during this experiment (p < 0.05). The data are shown in Table S1. For the shell size, both diameter and height increased gradually from 3.85 ± 0.69 cm to 5.50 ± 1.59 cm and from 2.19 ± 0.48 cm to 3.31 ± 0.88 cm, respectively (Fig. 1A). The body weight of sea urchins in July was 2.8 times heavier than that in January (from 17.68 ± 0.97 g to 48.79 ± 2.72 g), while the gonad weight in July was approximately 12 times heavier than that in January (from 0.70 ± 0.05 g to 8.37 ± 0.80 g) (Fig. 1B). We compared gonad somatic index (GSI) and found that GSI at the beginning of the experiment (3.98 ± 0.25%) was 4.3 times higher than that at the end point (17.16 ± 0.81%).

**Figure 2:**
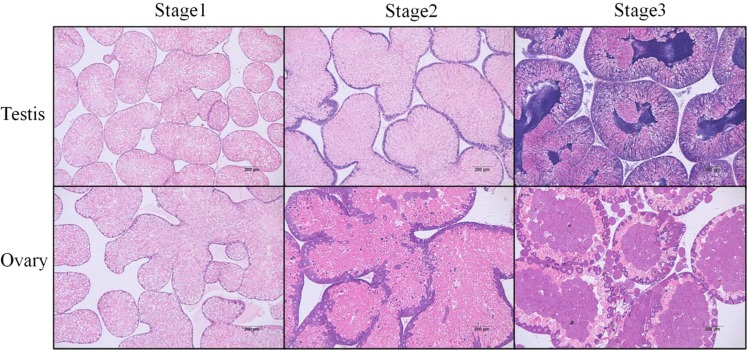
Histological features of the sea urchin *Strongylocentrotus intermedius* gonads at different gonad development stages. Paraffin-embedded sections were stained with hematoxylin and eosin (HE). Stage 1: A, D; stage 2: B, E; stage 3: C, F. Scale bar: 200 μm.

To separate the different sexes of sea urchins, all of the gonads of sea urchins used in this study were examined via microscopic observation (Fig. 2). The gonadal maturity and sex of each sea urchin were classified and determined as described by Unuma *et al*.^25^. In January, the gonads of both sexes were at the recovering stage (stage 1), and follicular lumina of the immature gonads were filled with nutritive phagocytes and a few scattered developing gonocytes. In April, the gonads were at the growing stage (stage 2), and spermatocytes or oocytes were assembled at the peripheral lines of follicular lumina. Then in July, the gonads developed to the reproductive stage (stage 3), and the ripe spermatozoa and ova assembled in the center of the follicular lumina. These gonads were selected for RNA and metabolite extraction.

### Metabolomic alterations during gonad development

**Figure 3:**
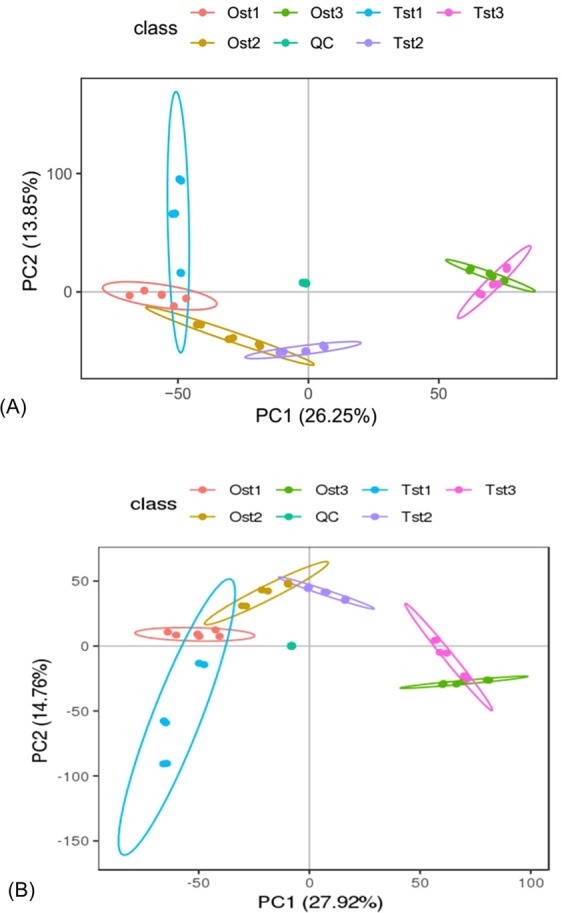
Principal component analysis (PCA) score plots of sea urchin gonads in positive and negative ion modes. Ost1: ovary at stage 1; Ost2: ovary at stage 2; Ost3: ovary at stage 3; Tst1: testis at stage 1; Tst2: testis at stage 2; Tst3: testis at stage 3; QC: quality control (n = 6).

The non-targeted metabolomic profile was assessed in our previous study (the paper is under review). To assess metabolomic alterations during gonad development, the principal component analysis (PCA) of sea urchin gonad development was generated from the LC-MS metabolite data of each gonadal development stage, the score plots are displayed in Fig. 3. In the score plots, each ellipse represents one group and each symbol corresponds to one sample. Remarkably, the six groups could be completely separated by the use of PCA. The first principle component (PC1, accounting for 26.25%) and the second component (PC2, accounting for 13.85%) of the variation in the data could separate all six groups with no outliers in the positive mode, meanwhile in the negative mode, PC1 and PC2 accounted for 27.92% and 14.76%, respectively.

### Differential expression and accumulation of genes and metabolites related to PUFA biosynthesis and metabolism

To investigate the mechanism of PUFA biosynthesis and metabolism in sea urchin gonad, integrated analysis was performed on the metabolic and transcript levels involving PUFA biosynthesis and metabolism. We found six differentially accumulated metabolites associated with PUFA, including arachidonic acid, docosapentaenoic acid, eicosapentaenoic acid, linoleic acid, punicic acid, and tetracosahexaenoic acid in the metabolomic analysis. The statistical data of DEGs related to PUFA are shown in Table 1. We focused on the DEGs related to PUFA in both sexes of sea urchin. By contrast, there were more DEGs related to PUFA in testis than ovaries (1823 DEGs in testis and 1499 DEGs in ovary). In particular, there were 1063 DEGs which were related to six PUFAs in the comparison of Tst1 and Tst3 (Tst1: testis at stage 1; Tst3: testis at stage 3).

**Table 1:** Descriptive statistics of the differentially expressed genes related to polyunsaturated fatty acid during gonad development.

Group	Mode	Differential metabolites	Number of DEGs
Ost1 vs Ost2	pos	Arachidonic acid	64
		Linoleic acid	39
	neg	Tetracosahexaenoic acid	65
Ost1 vs Ost3	pos	Arachidonic acid	89
		Eicosapentaenoic acid	69
		Linoleic acid	187
		Punicic acid	148
	neg	Docosapentaenoic acid	169
		Tetracosahexaenoic acid	171
Ost2 vs Ost3	pos	Eicosapentaenoic acid	106
	neg	Docosapentaenoic acid	94
		Tetracosahexaenoic acid	129
Tst1 vs Tst2	pos	Arachidonic acid	61
		Linoleic acid	20
	neg	Docosapentaenoic acid	46
		Tetracosahexaenoic acid	30
Tst1 vs Tst3	pos	Arachidonic acid	259
		Linoleic acid	86
		Stearidonic acid	422
	neg	Docosapentaenoic acid	129
		Eicosapentaenoic acid	107
		Tetracosahexaenoic acid	60
Tst2 vs Tst3	pos	Linoleic acid	195
	neg	Docosapentaenoic acid	112

Note: DEGs: differentially expressed genes; Ost1: ovary at stage 1; Ost2: ovary at stage 2; Ost3: ovary at stage 3; Tst1: testis at stage 1; Tst2: testis at stage 2; Tst3: testis at stage 3.

**Figure 4:**
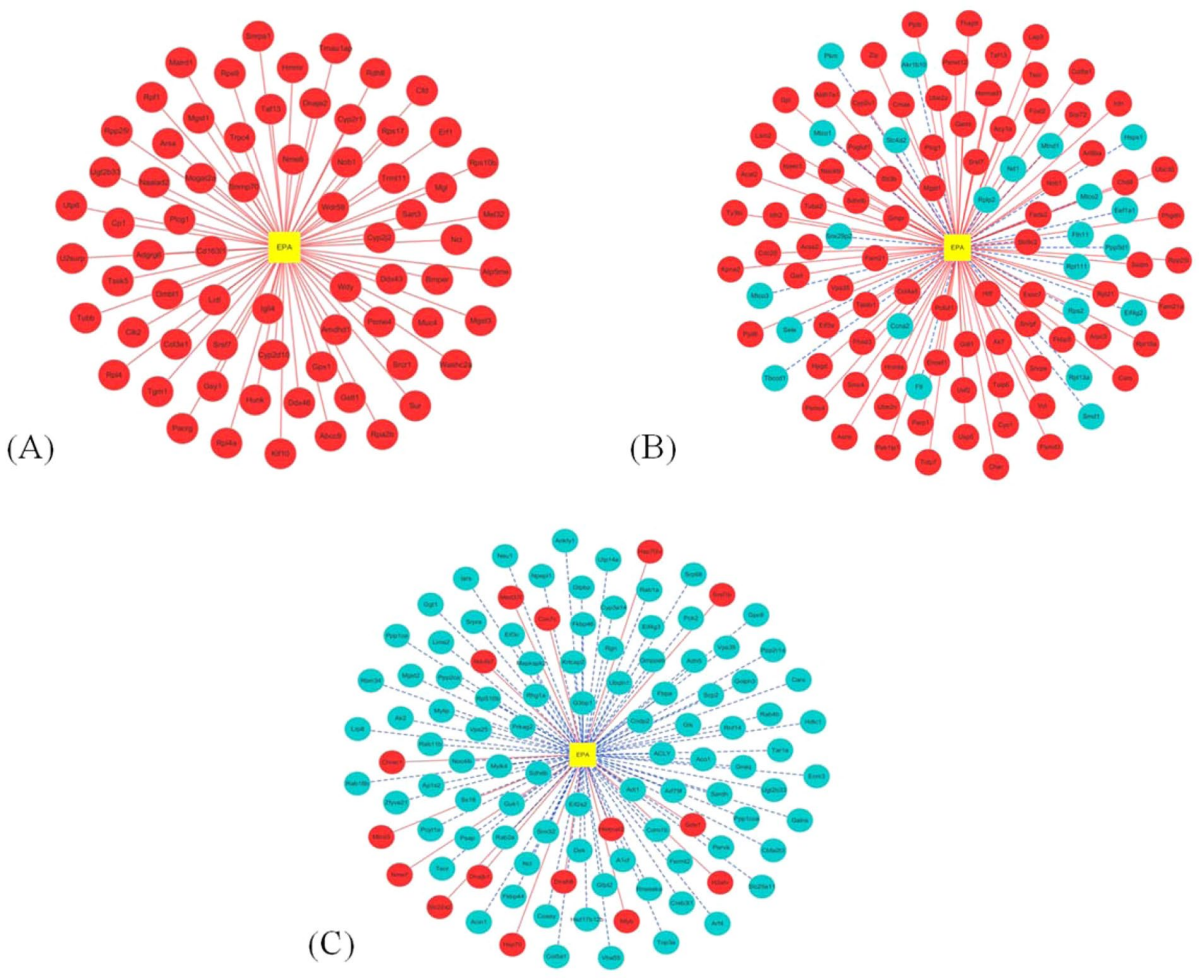
The differentially expressed genes (DEGs) related to eicosapentaenoic acid (EPA) during the gonad development of *Strongylocentrotus intermedius*. The DEGs are detected in three groups: Ost1 vs Ost3 (**A**), Ost2 vs Ost3 (**B**), and Tst1 vs Tst3 (**C**), respectively. Ost1: ovary at stage 1; Ost2: ovary at stage 2; Ost3: ovary at stage 3; Tst1: testis at stage 1; Tst3: testis at stage 3. The DEGs in the red circles represent upregulated genes and the DEGs in the blue circle represent downregulated genes.

In this study, specific DEGs related to eicosapentaenoic acid (EPA) in sea urchin gonad were selected (Fig. 4). We found that there were 69, 106 and 107 DEGs related to EPA in the groups Ost1 vs Ost3, Ost2 vs Ost3 (Ost1: ovary at stage 1; Ost2: ovary at stage 2; Ost3: ovary at stage 3) and Tst1 vs Tst3, respectively. All of these DEGs were assessed by Gene Ontology (GO) analysis and the evolutionary genealogy of genes: Non-supervised Orthologous Groups (eggNOG) database to predict and classify their possible biological functions in Supplemental Figure S2 and S3, respectively. The most abundant function groups were extracellular exosome, intracellular signal transduction, integral component of membrane steroid hydroxylase activity, oxidation-reduction process, hydrogen ion transmembrane transport, and cytosolic large ribosomal subunit in the GO analysis. In addition, “posttranslational modification, protein turnover, chaperones”, “translation, ribosomal structure, and biogenesis”, and “energy production and conversion” were the three major groups associated with the DEGs related to EPA in the eggNOG database.

**Figure 5:**
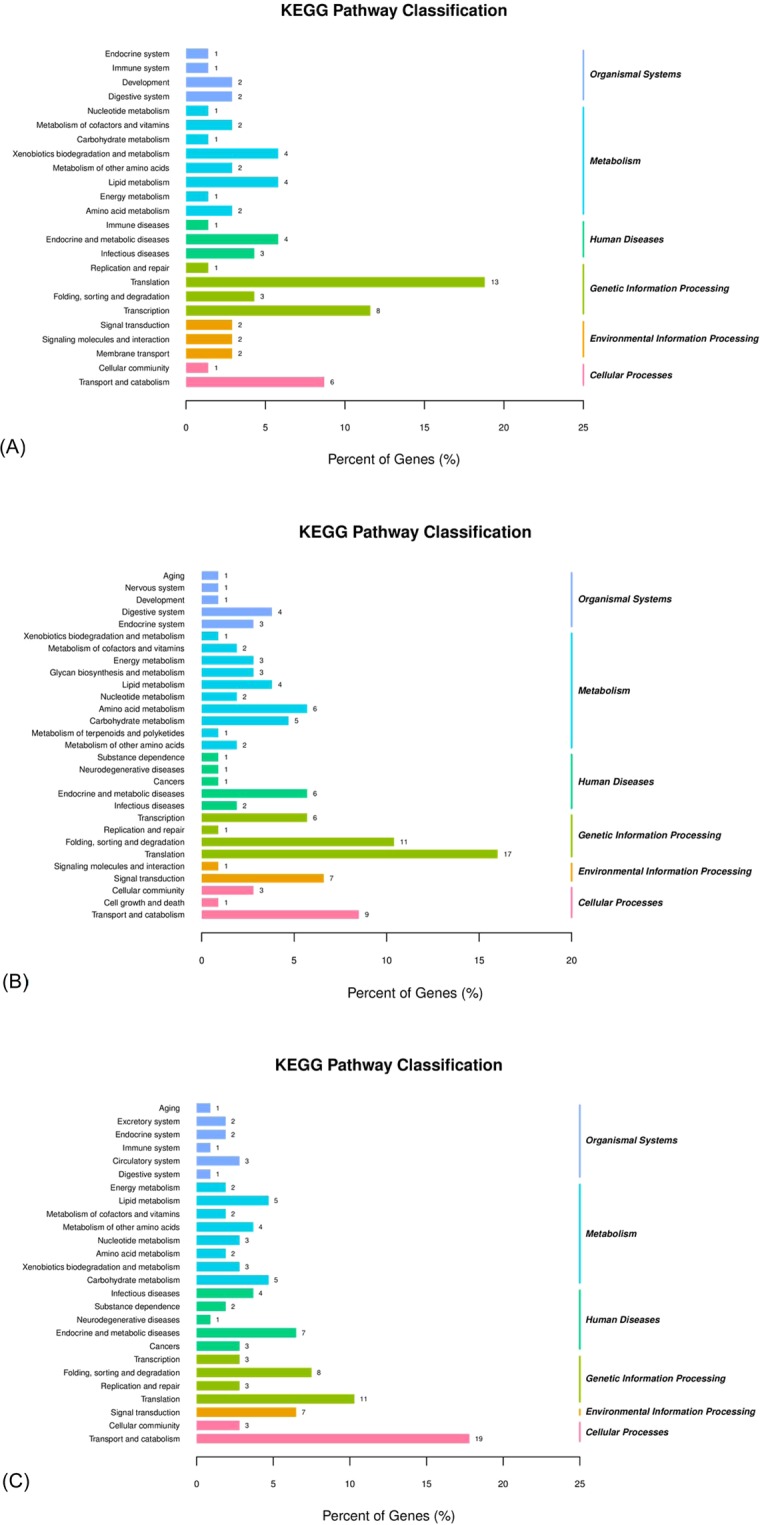
Functional classification of the differentially expressed genes (DEGs) in *Strongylocentrotus intermedius* by using Kyoto Encyclopedia of Genes and Genomes (KEGG) terms. The DEGs are detected in three groups: Ost1 vs Ost3 (**A**), Ost2 vs Ost3 (**B**), and Tst1 vs Tst3 (**C**), respectively. Ost1: ovary at stage 1; Ost2: ovary at stage 2; Ost3: ovary at stage 3; Tst1: testis at stage 1; Tst3: testis at stage 3.

To further elucidate the physiological implications and interactions of the genes identified in our data, we also blasted the DEGs relating to EPA in the Kyoto Encyclopedia of Genes and Genomes (KEGG) database (Fig. 5). In female sea urchins, DEGs related to translation, transcription, the process of folding, sorting and degradation, the process of transport and catabolism, and transport and catabolism were highly presented in the groups Ost1 vs Ost2 and Ost2 vs Ost3. In total, there were 46 and 60 DEGs assigned to the categories of metabolism and genetic information processing, respectively. Moreover, in the Tst1 vs Tst3 group of male sea urchins, 22, 25, and 26 DEGs were assigned to the categories of cellular processes, genetic information processing, and metabolism, respectively. Among these pathways, genes related to transport and catabolism, translation, and the process of folding, sorting and degradation were highly presented in this set of DEGs.

### Analysis for verification of the RNA-Seq results

To validate the transcription profile revealed by RNA-Seq data in each gonad development stage, qRT-PCR analysis was performed in 12 DEGs (Acat2, Adh5, Aldh7a1, Cyp2j2, Cyp2r1, Cyp2u1, Cyp3a14, Ecm3, Fads2, Gpx1, Hsd17d12, and Tecr genes) related to EPA in the integrated analysis. The expression patterns of all these genes in different development stages qualified by qRT-PCR coincided with the result of the transcriptomic sequencing, as shown in Fig. 5. This demonstrated the accuracy of our method, which detected the correct direction of gene expression change in all of the genes.

In male sea urchins, the expression levels of seven genes including Adh5, Cyp2j2, Cyp2r1, Cyp2u1, Cyp3a14, Ecm3, and Fads2 decreased gradually with gonad growth and development. By contrast, in the female sea urchins, the expression level of Cyp2j2, Cyp2r1, Cyp2u1, Cyp3a14, and Tecr decreased gradually. We found the expression level of Adh5 and Gpx1 in the ovary were 5–10 times higher than those in the testis. Moreover, Aldh7a1, Ecm3, Fads, and Hsd17b12 showed the highest expression levels in stage 2 ovaries.

## Discussion

Understanding the mechanism of PUFA biosynthesis and metabolism in sea urchin might be the first step toward selecting an economic sea urchin species, expressing their utmost potential to improve the quality of the gonad, and finally helping the sea urchin aquaculture to achieve sustainable development. Transcriptome sequencing technology has been widely used in exploring the critical metabolic pathways and functional genes that are involved in PUFA biosynthesis and metabolism in some species, even those that lack reference genomes. In our previous studies, we analyzed transcriptomic and metabolomic data separately^26,27^. Therefore, the aim of the present study was to integrate these two methods and to select the critical genes which were more associated with PUFAs during sea urchin gonad growth and development.

**Figure 6:**
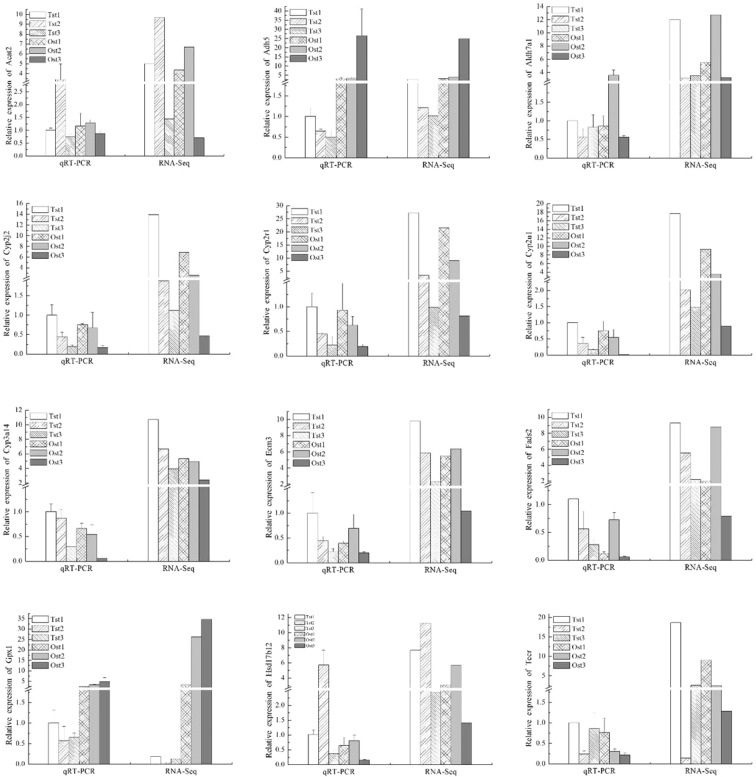
Quantitative (q)RT-PCR validation of the genes related to eicosapentaenoic acid (EPA) were differentially expressed among the different sexes and different development stages of the gonads. The relative expression of each gene were normalized to 18 S rRNA gene. Bars represent the mean ± SEM (n = 3). Ost1: ovary at stage 1; Ost2: ovary at stage 2; Ost3: ovary at stage 3; Tst1: testis at stage 1; Tst2: testis at stage 2;Tst3: testis at stage 3.

PUFA biosynthesis and metabolism processes involve multiple gene expression networks and complex biochemical pathways. As shown in Table 1, there were hundreds of DEGs related to six PUFAs. Therefore, we selected and focused on EPA, which was the main component of PUFAs in *S. intermedius* gonads. As shown in Supplemental Figure S1, in the ovary, the EPA concentration at stage 2 was increased by three times that at stage 1 (p < 0.05). However, there were no significant changes in testis (p > 0.05). Here, 12 genes were isolated from DEGs. In this study, the results of qRT-PCR confirmed that Aldh7a1, Ecm3, Fads2, and Hsd17b12 had similar expression patterns with EPA concentration, which are presented in Fig. 6. This evidence indicated that the upregulated expression of these genes during gonad growth and development might be controlled by the above transcription factors, and these genes might control EPA biosynthesis. In general, alpha-aminoadipic semialdehyde dehydrogenase (Aldh7a1), encoded by the Aldh7a1 gene in mammals, involved in lysine catabolism, is a multifunctional enzyme mediating important protective effects. It can metabolize betaine aldehyde to betaine, an important cellular osmolyte, and methyl donor. Moreover, it could protect cells from oxidative stress by metabolizing a number of lipid peroxidation-derived aldehydes^28^. Extracellular matrix protein 3 (Ecm3), in the green sea urchin *Lytechinus variegatus*, may serve as a substrate for the migratory primary mesenchyme cells (PMCs), the interaction possibly providing guidance to migrating PMCs^29^. Zhao *et al*.^30^ reported that there was down-regulation in fatty acid oxidation (FAO) in normal skin with abundant ECM, and FAO pathway enzymes revealing their reciprocal effects in ECM down-regulation. Fatty acid desaturase 2 (Fads2), encoded by the Fads2 gene is a member of the fatty acid desaturase gene family with both Delta5 and Delta6 activities, and is also known as Delta5/Delta6 fatty acid desaturase in vertebrates. This protein is involved in the pathway of PUFA biosynthesis, which is involved in lipid metabolism. Fads2 catalyzes the first and rate-limiting step in this pathway which is the desaturation of LA (18:2n-6) and a-linolenic acid (18:3n-3, ALA) into gamma-linoleate (18:3n-6) and stearidonate (18:4n-3, SDA), respectively^31^. Subsequently, in the biosynthetic pathway of HUFA n-3 series, desaturates tetracosapentaenoate (24:5n-3) to tetracosahexaenoate (24:6n-3), which is then converted to docosahexaenoate (DHA) (22:6n-3)^32^. However, Fads2 did not exhibit Delta5-desaturase activity with the substrates dihomo-γ-linolenic acid (20:3n-6) or eicosatetraenoic acid (20:4n-3)^33^. Very long-chain 3-oxoacyl-CoA reductase (Hsd17b12) catalyzes the second of the four reactions of the long-chain fatty acids elongation cycle, and this enzyme has 3-ketoacyl-CoA reductase activity, reducing 3-ketoacyl-CoA to 3-hydroxyacyl-CoA, within each cycle of fatty acid elongation. It may participate in the production of PUFAs of different chain lengths that are involved in multiple biological processes as precursors of membrane lipids and lipid mediators^34^, especially like EPA, which may potentially drive myoblasts to enter the quiescent state and enable adipogenic trans-differentiation of the myoblasts and impairs brown adipogenesis and thermogenic capacity^35,36^.

As shown in Fig. 6, there were several down-regulation genes such as Adh5, Cyp2j2, Cyp2r1, Cyp2u1, Cyp3a14, Ecm3, and Fads2 in testis, as well as Cyp2j2, Cyp2r1, Cyp2u1, Cyp3a14, and Tecr in ovary. Their expression level decreased gradually with gonad growth and development. However, EPA concentration did not show a significant decrease in the gonads of both sexes, on the contrary, EPA increased at stage 2 in ovary. This might indicate that sea urchin needs to accumulate more PUFA such as EPA in the gonad with gonad growth and development, especially in females, for spawning during the mature period. Sex was considered as the main factor affecting fatty acid profile in the gonads of sea urchin *Arbacia dufresnii*^37^, and these differences might also affect the spawning and egg quality of Chinese prawn *Penaeus chinensis*^38,39^. Cyp2j2, Cyp2u1, and Cyp3a14 are members of the cytochrome P450 superfamily of oxygenases, which catalyze many reactions involved in the metabolism of drugs and other xenobiotics, as well as in the synthesis of cholesterol, steroids, and the hydroxylation of fatty acids and fatty acid metabolites. Cyp2j2 is thought to be a prominent enzyme responsible for metabolizing endogenous PUFA to signaling molecules. It metabolizes endogenous arachidonic acid (AA) to eicosatrienoic acid epoxides^40^, and also metabolizes EPA to various epoxyeicosate traenoic acids^41^. Cyp2u1 metabolizes long-chain fatty acids, such as AA and DHA, which suggests that it may play a role in brain and immune functions. It may modulate the AA signaling pathway and participates in the signaling processes of other fatty acids^42^. Cyp3a14 is known to be involved in the NADPH-dependent electron transport pathway, and it oxidizes various structurally unrelated compounds, such as fatty acids, xenobiotics, and steroids in liver microsomes of mammals. Alcohol dehydrogenase class-3 encoded by the Adh5 gene is remarkably ineffective in oxidizing ethanol, but it readily catalyzes the oxidation of S-(hydroxymethyl) glutathione and of long-chain primary alcohols^43^. The Tecr gene encodes very long-chain enoyl-CoA reductase which is also involved in both the production of very long-chain fatty acids for sphingolipid synthesis and the degradation of the sphingosine moiety in sphingolipids through the sphingosine 1-phosphate metabolic pathway^44^. We have inferred that the expression of these genes might be decreased to reduce EPA or PUFA metabolized as energy during these periods.

The PUFA biosynthesis and metabolism during gonad growth and development in the sea urchin *S. intermedius* are two very complex and dynamic processes. Many candidate genes have been identified in this work, which could be helpful in understanding these characteristics and their regulatory networks. However, further analysis of the promoter and regulatory elements as well as the functions of these genes might enable better definition of the molecular mechanism of fatty acid biosynthesis and metabolism in sea urchin in our future studies.

## Materials and Methods

### Animals and sample preparation

Adult *S. intermedius* sea urchins were collected in December 2017 in Dalian, China. After collection, we fed sea urchins with the kelp *Laminaria japonica* in the lab of Dalian Ocean University, China, and the feeding experimental period was from January 2018 to July 2018.

The gonad samples were collected every three months. Before dissection, we measured the growth index, such as shell diameter, shell height, body weight and gonad weight of each individual. Several small pieces of gonads were flash-frozen in liquid nitrogen for metabolomic and transcriptomic analysis and other pieces were fixed in Bouin’s solution for histological observation. We prepared the paraffin sections with thickness of 6 μm and stained them with hematoxylin/eosin (HE; Beyotime Institute of Biology, Suzhou, China).

### Transcriptomic analysis

Transcriptomic analysis was carried out during the gonadal development of both sexes of sea urchins, and we selected three males and three females from each gonadal development stage. The total RNA of each sample were extracted, quantity and purity checked, and sequeced by the Illumina Hiseq. 4000 (LC-Bio Tchnologies, Hangzhou, China) following the manufacturer’s protocol, while the details were decribed in our pervious study^26^.

After sequencing, de novo assembly of clean reads, unigene annotation and functional classification were performed and the DEGs were selected by the edgeR package^45^. All raw sequence files and details have been deposited in the NCBI Sequence Read Archive database under accession number PRJNA532998. The DEGs related to EPA were selected and annotated by a sequence alignment similarity search in this work. GO annotation (http://www.geneontology.org), eggNOG (http://eggnogdb.embl.de/), and KEGG (http://www.genome.jp/kegg/) databases were performed for functional annotation^46^.

### Quantitative real-time polymerase chain reaction analysis

To validate the transcriptomic sequencing data, quantitative real-time polymerase chain reaction (qRT-PCR) was applied, and 12 DEGs were selected using the same samples as for the transcriptomic sequencing. The analysis was conducted with a LightCycler® 96 instrument (Roche Life Science, Basel, Switzerland), and gene-specific primers were designed using Primer Premier 5.0 (Supplemental Table S2). The detailed processes of cDNA reverse transcription, gene amplification and internal standardization were decribed in our pervious study^47^. Three independent biological replicates of each gender at each development stage were performed to increase the veracity of the results. The 18 S ribosomal RNA gene (*18 S rRNA*) was used as the housekeeping gene for internal standardization in this study.

### Metabolomic and metabolite analysis

Six males and six females from each gonadal development stage of sea urchin were selected and collected for metabolites extraction. The metabolomic analysis was carried out using an ultra-performance liquid chromatography (UPLC) system (SCIEX, Warrington, UK) following the manufacturer’s protocol in LC-Bio Tchnologies (Hangzhou) Co., Ltd. The detailed processes of metabolies annotation and identification analysis were decribed in our pervious study^27^.

### Data analysis

The analysis methods included PCA and partial least-squares discriminant analysis (PLS-DA). Supervised PLS-DA was conducted through metaX to discriminate the different variables between groups. The variable important for the projection (VIP) value was a weighted sum of squares of the PLS weights, and the variables with VIP ≥ 1; ratio ≥ 2 or ratio ≤ 1/2; q value ≤ 0.05 were considered to be influential for the separation of the samples in the score plots generated from PLS-DA.

From RNA-seq and LC-MS/MS results, the DEGs and modified metabolites were used to identify the biological pathway by using KEGG and GO^48,49^, and data were converted into vectors to calculate Pearson’s correlation coefficient between metabolite-metabolite, gene-gene, and metabolite-gene profiles.

Statistical analysis involved the use of SPSS software version 19.0 (IBM, Armonk, NY, USA). The results are expressed as the mean ± SEM. Significant differences (p < 0.05) for each variable were first detected using the one-way ANOVA test, followed by Tukey’s HSD.

## Supplementary information

Supplementary Information.
