# Supplementary material for: Integrated metabolomic and transcriptomic analyses identify critical genes in eicosapentaenoic acid biosynthesis and metabolism in the sea urchin Strongylocentrotus intermedius
Source: Sci Rep. 2020 Feb 3;10:1697. doi: 10.1038/s41598-020-58643-x (PMC6997175; doi:10.1038/s41598-020-58643-x)
Supplement: Supplementary file 1 — Supplementary Information. [file 41598_2020_58643_MOESM1_ESM.pdf]

Integrated metabolomic and transcriptomic analyses identify critical genes in eicosapentaenoic acid biosynthesis and metabolism in the sea urchin *Strongylocentrotus intermedius*

Heng Wang [1, †], Jun Ding [1, †,*], Siyu Ding [1], Yaqing Chang [1,*]

[1] Key Laboratory of Mariculture& Stock Enhancement in North China's Sea, Ministry of Agriculture and Rural Affairs, Dalian Ocean University, Dalian, 116023, China; hengwang@dlou.edu.cn (H. Wang); siyu19860608@163.com (S. Ding)

[#] Equal contributors

* Correspondence: dingjun19731119@hotmail.com (J. Ding);
changlab@hotmail.com (Y. Chang)

Tel.: +86-411-84762871

Supplementary Table S1 Biometrics of the sea urchin *Strongylocentrotus intermedius* sampled for RNA and metabolite extraction.

Stage	Shell diameter (cm)	Shell height (cm)	Wet body weight (g)	Wet gonad weight (g)	Gonado-somatic index (%)
1	3.85 ± 0.69	2.19 ± 0.48	17.68 ± 0.97	0.70 ± 0.05	3.98 ± 0.25
2	4.14 ± 0.94	2.22 ± 0.79	29.28 ± 1.56	3.67 ± 0.27	12.52 ± 0.50
3	5.50 ± 1.59	3.31 ± 0.88	48.79 ± 2.72	8.37 ± 0.80	17.16 ± 0.81

Supplemental Table S2 The primers used in this study

Gene	Forward primer (5'→3')	Reverse primer (5'→3')
Adh5	AGTGAATACACCGTCGTTGCTGAG	CACAGCCAGACCTACACATCCAAC
Aldh7a1	AGCCATAGCCAGAGTCAGACAGG	ATCTGCCTCACGACCTCACCTC
Acat2	TGGTTCCGGCCTGAGGACAATAG	AGGACAGACCGCTACGAAGATGG
Cyp2j2	AGATGGATCAGGTGGTCGGAAGAG	CGTCAGCATCAGCCATGTGAGG
Cyp2r1	CCTTCAAGAAGCCAGACCACTCAC	TGGAATAGCGCAGGTGAGGTAGG
Cyp2u1	GCCTCCGTCGCCTCCTAGTC	TTCGCTCATCACCACAATCCTCTG
Cyp3a14	CTCCGCTTGCCAATCGTCCTATG	ACCATGCTTCTCTTGCTCTTGTCC
Ecm3	TAACGGAATCGCCTTCAACGCTAC	GCCGATGCCAGATGTCTCAGATG
Fads2	ACTCGCACACGACTTCGATAACG	GCCAGGAGGAAGGAGAAGATGTTG
Gpx1	TTGCTACGATGACGCCGAATCC	GCAGAATGACGCCGTATTGACAAC
Hsd17b12	AGCTGACCGGACTGGACATCG	ACGACAGACAGGCAGTTGATGTTG
Tecr	CGCCACCATGCCTATCACCAAC	GGTCACGCAGGACAATGTGGATAC

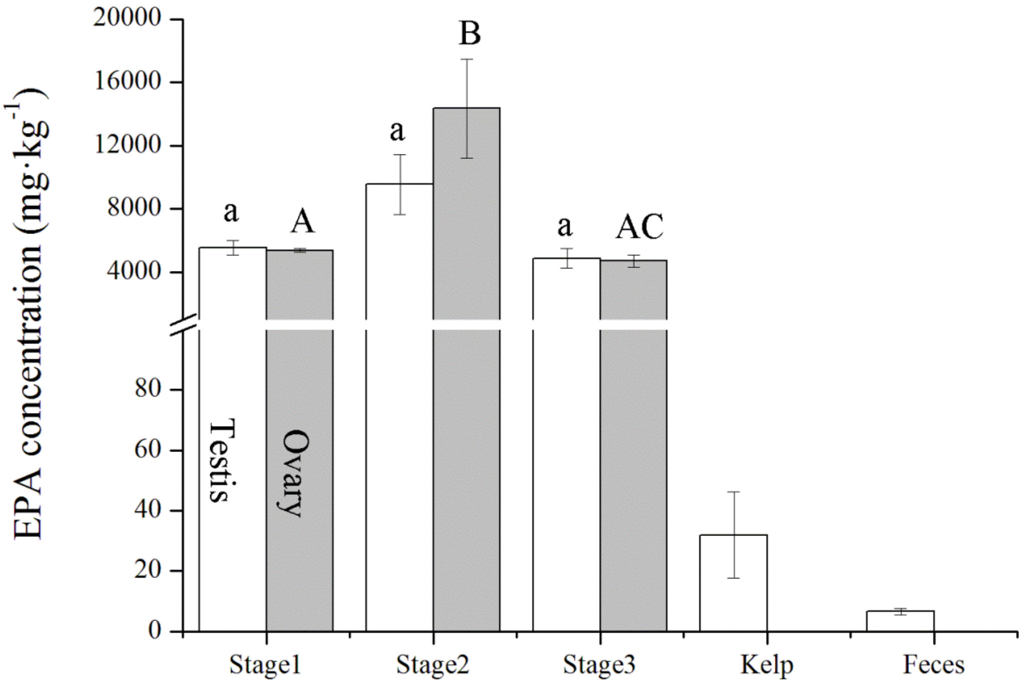

Supplemental Figure S1. Eicosapentaenoic acid (EPA) concentration in each developmental gonad of sea urchin, kelp, and feces. a, b, c (testis) and A, B, C (ovary) indicate significant differences among stages within each sex ($p < 0.05$).

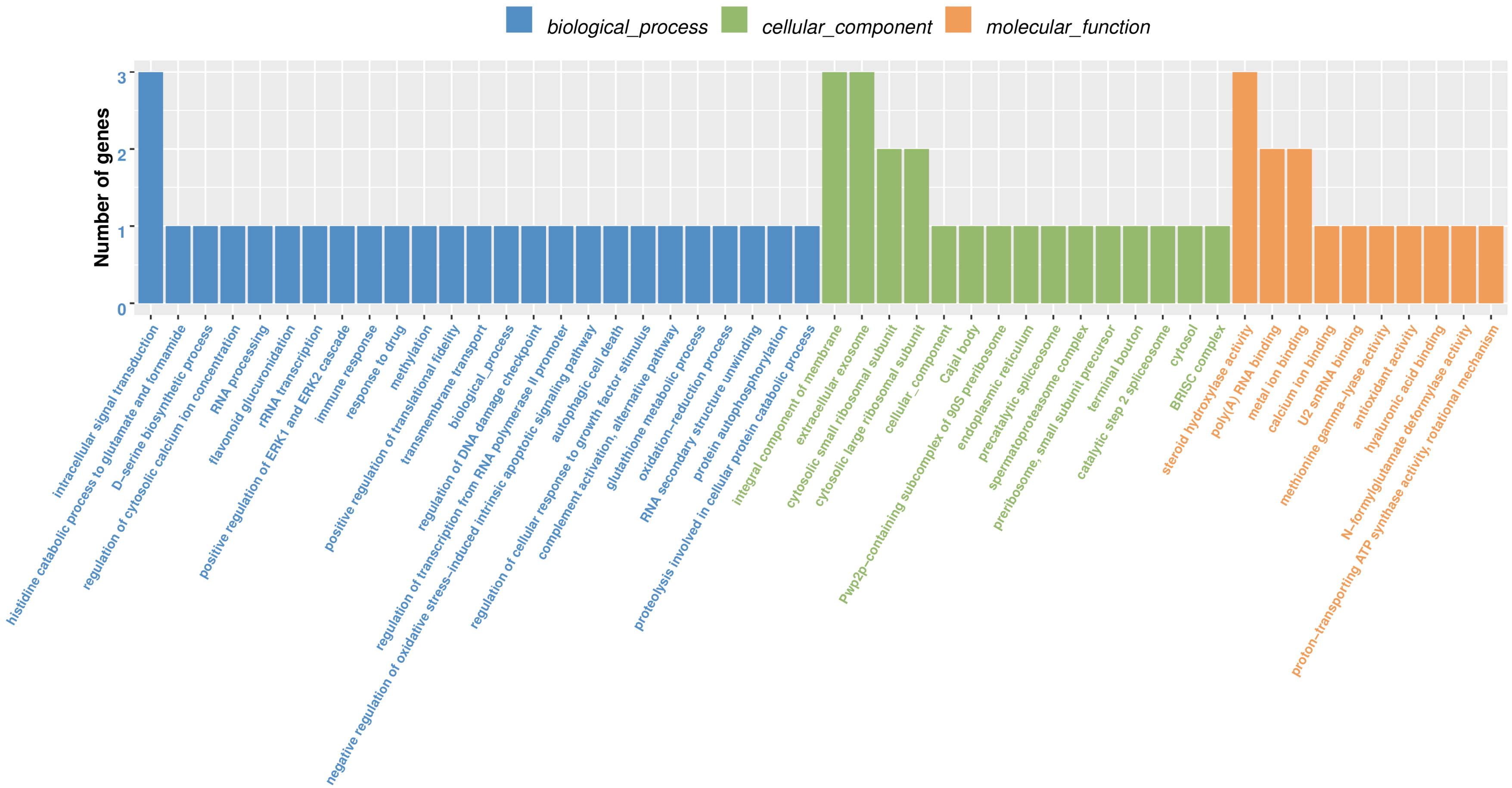
biological_process
cellular_component
molecular_function
Number of genes
0
1
2
3
intracellular signal transduction
histidine catabolic process to glutamate and formamide
D−serine biosynthetic process
regulation of cytosolic calcium ion concentration
RNA processing
flavonoid glucuronidation
rRNA transcription
positive regulation of ERK1 and ERK2 cascade
immune response
response to drug
methylation
positive regulation of translational fidelity
transmembrane transport
biological_process
regulation of DNA damage checkpoint
regulation of transcription from RNA polymerase II promoter
negative regulation of oxidative stress−induced intrinsic apoptotic signaling pathway
autophagic cell death
regulation of cellular response to growth factor stimulus
complement activation, alternative pathway
glutathione metabolic process
oxidation−reduction process
RNA secondary structure unwinding
protein autophosphorylation
proteolysis involved in cellular protein catabolic process
integral component of membrane
extracellular exosome
cytosolic small ribosomal subunit
cytosolic large ribosomal subunit
cellular_component
Cajal body
Pwp2p−containing subcomplex of 90S preribosome
endoplasmic reticulum
precatalytic spliceosome
spermatoproteasome complex
preribosome, small subunit precursor
terminal bouton
catalytic step 2 spliceosome
cytosol
BRISC complex
steroid hydroxylase activity
poly(A) RNA binding
metal ion binding
calcium ion binding
U2 snRNA binding
methionine gamma−lyase activity
antioxidant activity
hyaluronic acid binding
N−formylglutamate deformylase activity
proton−transporting ATP synthase activity, rotational mechanism

(A)

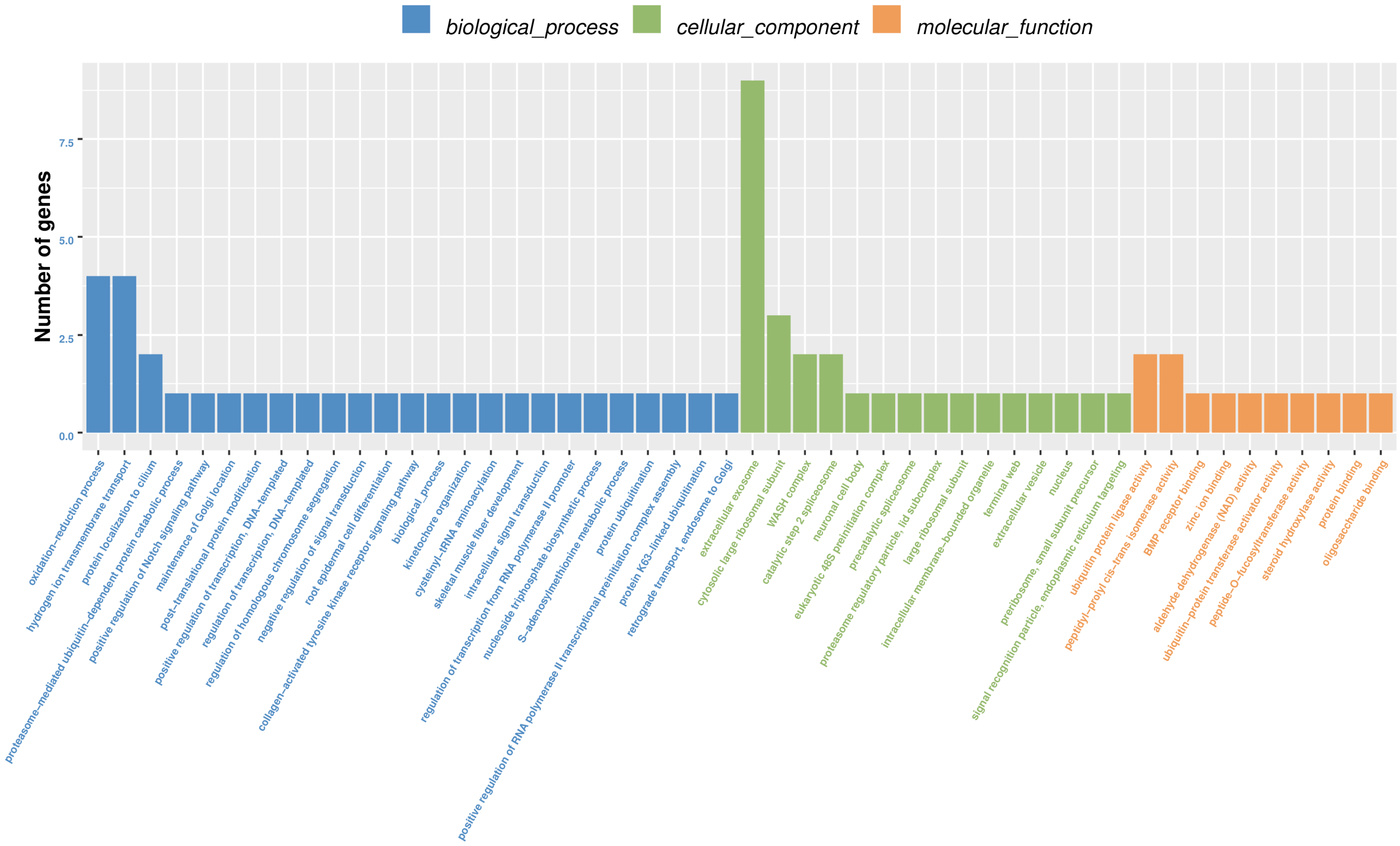

biological_process
cellular_component
molecular_function
Number of genes
0.0
2.5
5.0
7.5
oxidation–reduction process
hydrogen ion transmembrane transport
protein localization to cilium
proteasome–mediated ubiquitin–dependent protein catabolic process
positive regulation of Notch signaling pathway
maintenance of Golgi location
post–translational protein modification
positive regulation of transcription, DNA–templated
regulation of transcription, DNA–templated
regulation of homologous chromosome segregation
negative regulation of signal transduction
root epidermal cell differentiation
collagen–activated tyrosine kinase receptor signaling pathway
biological_process
kinetochore organization
cysteinyl–tRNA aminoacylation
skeletal muscle fiber development
intracellular signal transduction
regulation of transcription from RNA polymerase II promoter
nucleoside triphosphate biosynthetic process
S–adenosylmethionine metabolic process
protein ubiquitination
positive regulation of RNA polymerase II transcriptional preinitiation complex assembly
protein K63–linked ubiquitination
retrograde transport, endosome to Golgi
extracellular exosome
cytosolic large ribosomal subunit
WASH complex
catalytic step 2 spliceosome
neuronal cell body
eukaryotic 48S preinitiation complex
precatalytic spliceosome
proteasome regulatory particle, lid subcomplex
large ribosomal subunit
intracellular membrane–bounded organelle
terminal web
extracellular vesicle
nucleus
preribosome, small subunit precursor
signal recognition particle, endoplasmic reticulum targeting
ubiquitin protein ligase activity
peptidyl–prolyl cis–trans isomerase activity
BMP receptor binding
zinc ion binding
aldehyde dehydrogenase (NAD) activity
ubiquitin–protein transferase activator activity
peptide–O–fucosyltransferase activity
steroid hydroxylase activity
protein binding
oligosaccharide binding

(B)

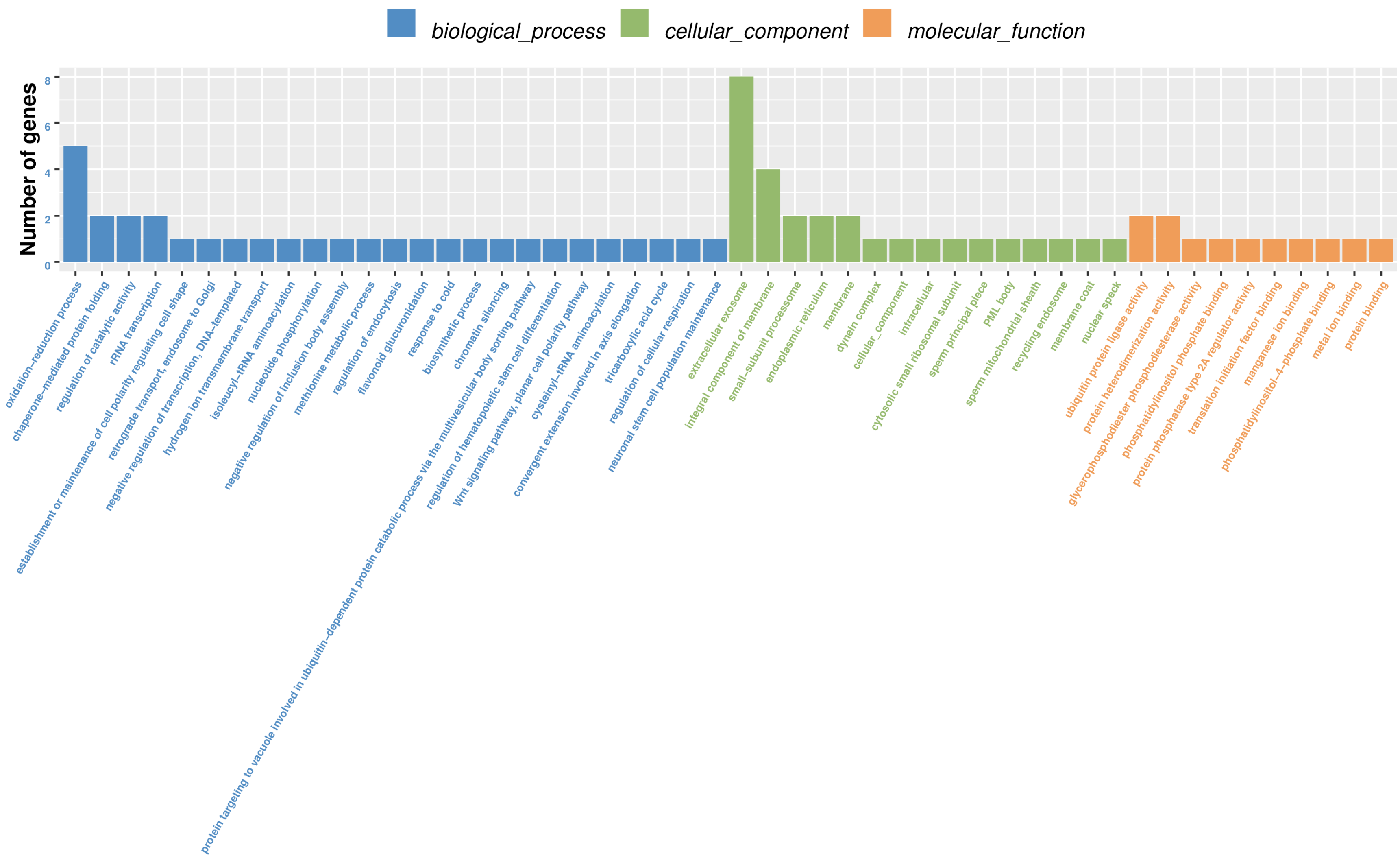

(C)

Supplemental Figure S2. Gene Ontology (GO) annotation of *Strongylocentrotus intermedius* genes

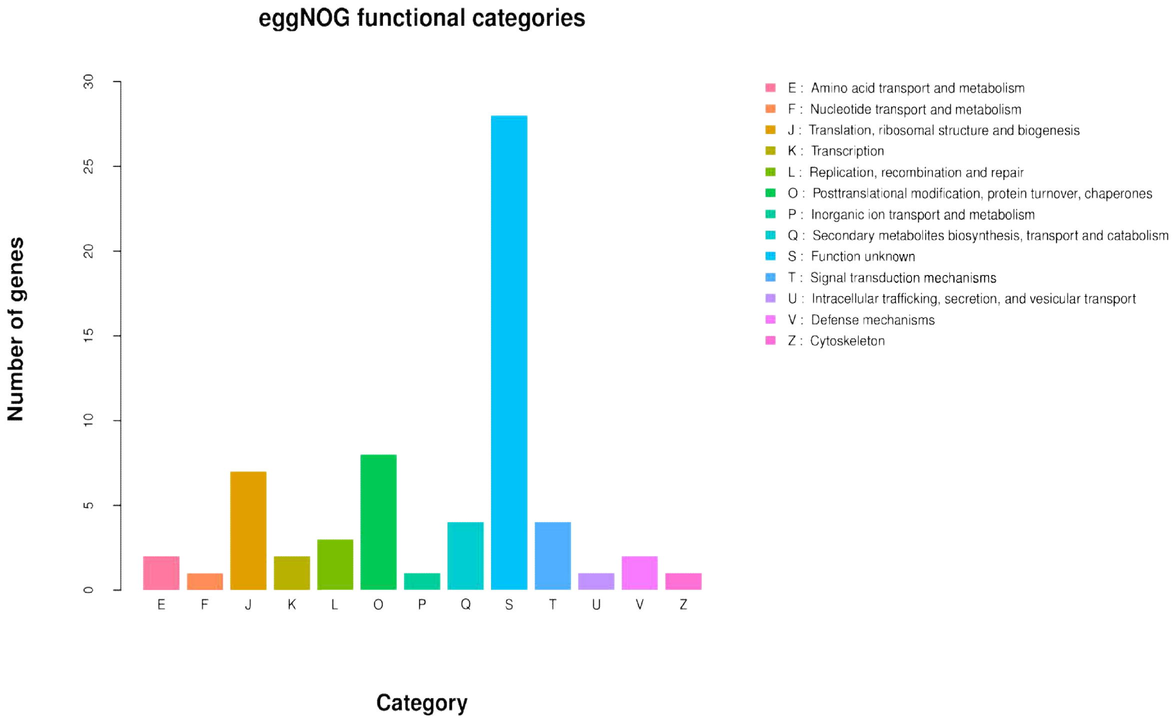
eggNOG functional categories
Number of genes
0
5
10
15
20
25
30
E
F
J
K
L
O
P
Q
S
T
U
V
Z
Category
E : Amino acid transport and metabolism
F : Nucleotide transport and metabolism
J : Translation, ribosomal structure and biogenesis
K : Transcription
L : Replication, recombination and repair
O : Posttranslational modification, protein turnover, chaperones
P : Inorganic ion transport and metabolism
Q : Secondary metabolites biosynthesis, transport and catabolism
S : Function unknown
T : Signal transduction mechanisms
U : Intracellular trafficking, secretion, and vesicular transport
V : Defense mechanisms
Z : Cytoskeleton

(A)

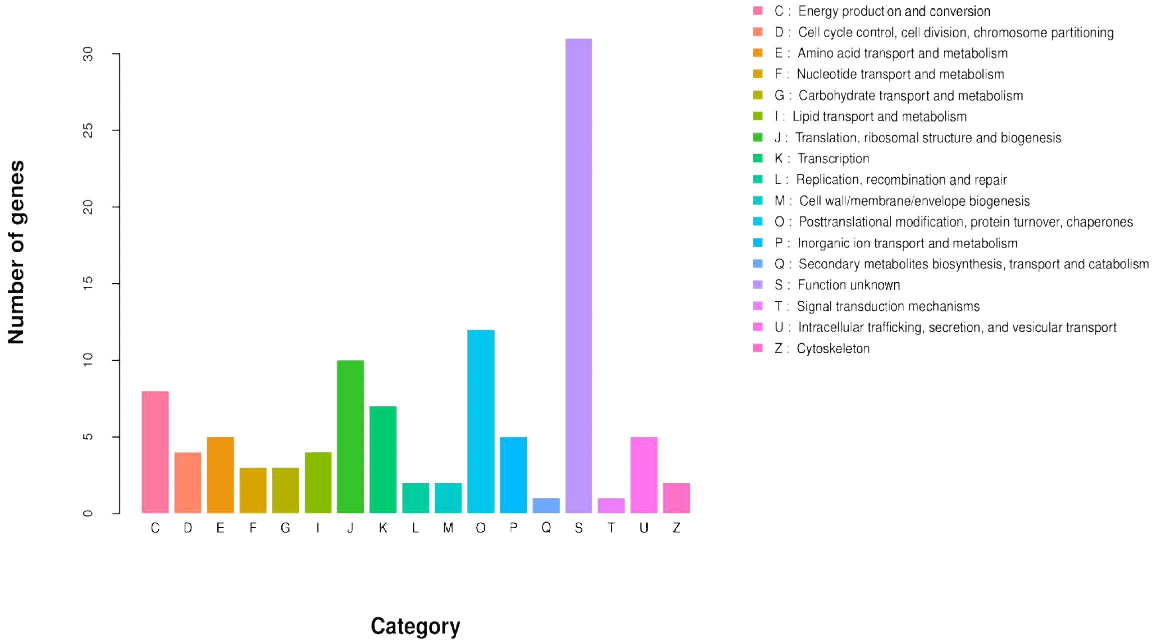
Number of genes
0
5
10
15
20
25
30
C
D
E
F
G
I
J
K
L
M
O
P
Q
S
T
U
Z
Category
C : Energy production and conversion
D : Cell cycle control, cell division, chromosome partitioning
E : Amino acid transport and metabolism
F : Nucleotide transport and metabolism
G : Carbohydrate transport and metabolism
I : Lipid transport and metabolism
J : Translation, ribosomal structure and biogenesis
K : Transcription
L : Replication, recombination and repair
M : Cell wall/membrane/envelope biogenesis
O : Posttranslational modification, protein turnover, chaperones
P : Inorganic ion transport and metabolism
Q : Secondary metabolites biosynthesis, transport and catabolism
S : Function unknown
T : Signal transduction mechanisms
U : Intracellular trafficking, secretion, and vesicular transport
Z : Cytoskeleton

(B)

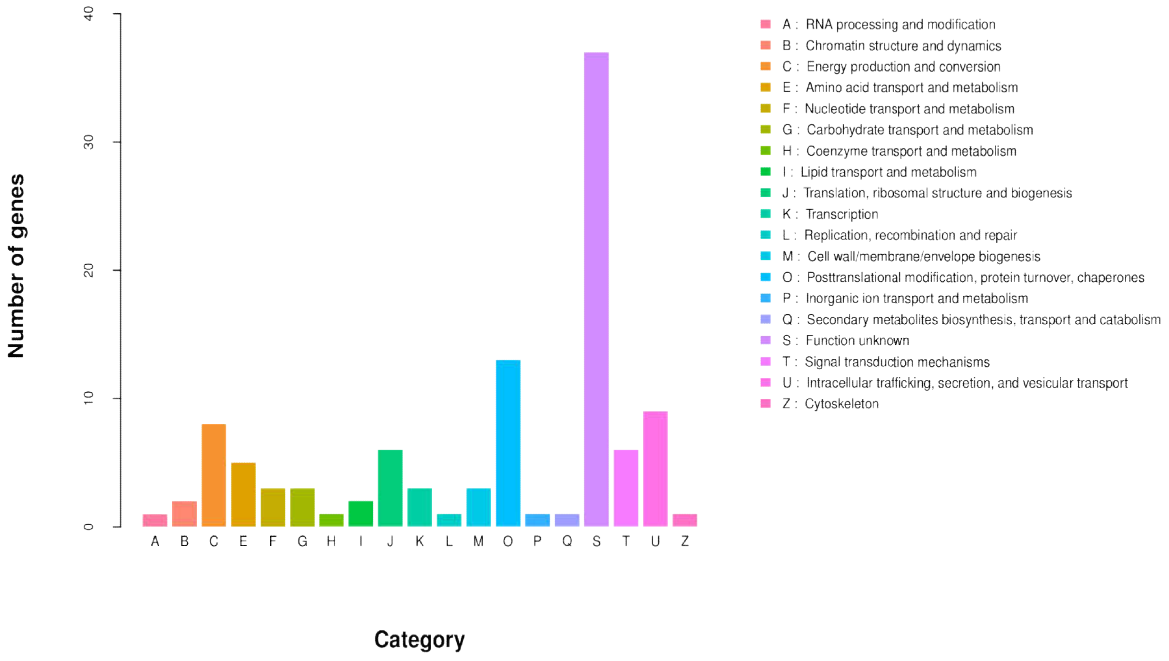

(C)

Supplemental Figure S3. Evolutionary genealogy of genes: Non-supervised Orthologous Group functional classifications of *Strongylocentrotus intermedius*.
